# Efficacy and safety of AZD3199 vs formoterol in COPD: a randomized, double-blind study

**DOI:** 10.1186/1465-9921-14-64

**Published:** 2013-06-03

**Authors:** Piotr Kuna, Yavor Ivanov, Vasily Ivanovich Trofimov, Takefumi Saito, Ola Beckman, Thomas Bengtsson, Carin Jorup, François Maltais

**Affiliations:** 1 Barlicki University Hospital, Medical University of Lodz, Kopcińskiego 22, Lodz, 90-153 Łódź, ul., Poland; 2 Medical University Pleven, Pleven, Bulgaria; 3 Pavlov State Medical University, St. Petersburg, Russia; 4 National Hospital Organization Ibaraki-Higashi National Hospital, Naka-gun, Japan; 5 AstraZeneca R&D, Mölndal, Sweden; 6 Institut Universitaire de cardiologie et de pneumologie de Québec, Université Laval, Québec, Canada

**Keywords:** Bronchodilation, β_2_-agonist, COPD, Once-daily, Ultra long-acting

## Abstract

**Background:**

We investigated the efficacy and safety of AZD3199, a novel inhaled ultra-LABA, with the main aim of establishing a dose that would maintain 24-hour bronchodilation in patients with COPD.

**Methods:**

Patients (n = 329) were randomized to AZD3199 (200, 400 or 800 μg o.d.), formoterol (9 μg b.i.d.) or placebo via Turbuhaler® in a parallel group study. The primary objective of the study was to compare the clinical efficacy of three doses of AZD3199 inhaled once daily with 9 μg formoterol twice daily and placebo, over a 4-week treatment period in adults with moderate-to-severe COPD. After 4 weeks, peak (0–4 h) and trough (24–26 h) forced expiratory volume in 1 second (FEV_1_) were assessed as the primary efficacy outcome variables.

**Results:**

All AZD3199 doses significantly increased mean peak and trough FEV_1_ versus placebo (106–171 ml and 97–110 ml increases, respectively), but with no clear dose–response; the level of bronchodilation was comparable to or greater than that achieved with formoterol. Forced vital capacity (FVC) at peak bronchodilation also significantly increased with AZD3199 versus placebo (153–204 ml). COPD symptom scores and reliever use were reduced with AZD3199, while FEV_1_ reversibility was unaltered. Adverse events were mild-to-moderate, with no safety concerns identified. Drug exposure was dose-proportional, but lower than predicted from healthy volunteers.

**Conclusions:**

All three doses of AZD3199 produced 24-hour bronchodilation, but with no clear dose–response, suggesting that doses of 200 μg or less may be sufficient to maintain bronchodilation over 24 hours in patients with COPD. No safety concerns were identified. Further studies are required to determine the once-daily AZD3199 dose for COPD.

**Trial registration:**

Clinicaltrials.gov, NCT00929708

## Background

Chronic obstructive pulmonary disease (COPD) is characterized by the progressive development of airway obstruction and airflow limitation. The condition is not fully reversible and typically leads to a decline in exercise performance and a deterioration in health [1]. COPD is associated with considerable morbidity and mortality, posing an enormous burden to the health care system [1,2]. Despite a decrease in the prevalence of smoking in developed countries, it is predicted that COPD will be the third leading cause of death worldwide by 2020 [3-5].

Symptomatic treatment with bronchodilators is recommended as the first stage of therapy for COPD [6]. As the symptoms of COPD require continual treatment, long-acting bronchodilators are more convenient for the patient and more effective on a number of endpoints than short-acting alternatives. Current guidelines for the treatment of COPD recommend a stepwise approach to treatment, with bronchodilators central to symptomatic management [6,7]. Regular use of long-acting β-agonists (LABA) and long-acting muscarinic antagonists (LAMA), either as monotherapy or in combination, is recommended to achieve optimal bronchodilation and improve health status [6-8].

AZD3199 is a novel inhaled, selective ultra-LABA (uLABA, i.e. ≥ 24-hour duration of action) that has been investigated in asthma and COPD. A Phase II trial in patients with asthma (NCT00736489) demonstrated AZD3199 to be a potent LABA, with a bronchodilatory effect that persisted over 24 hours (data on file). The aim of the present study was to compare the efficacy of three different doses of inhaled, once-daily AZD3199 with that of twice-daily formoterol 9 μg in patients with moderate to severe COPD. This study was conducted to establish an appropriate dose of AZD3199 to be used once-daily in patients with COPD.

## Methods

### Study design and medications

The trial was a 4-week randomized, double-blind, placebo-controlled, parallel-group study conducted at 53 sites in Bulgaria, Canada, Japan, Poland and Russia. Patients with moderate-to-severe COPD who were symptomatic during a 2-week run-in period (total COPD symptom score ≥2 for at least half the number of days of the run-in period) were randomized to treatment.

Patients who fulfilled the inclusion criteria and none of the exclusion criteria entered the 2-week run-in period with salbutamol as reliever medication (and inhaled glucocorticosteroids [GCS] at a constant dose, if applicable) as the only allowed COPD medications. Patients using an inhaled GCS combination product at entry were switched to the corresponding monoproduct. Patients had to have been treated with inhaled GCS for at least 30 days before study entry to be allowed to continue monotherapy during the study.

**Figure 1:**
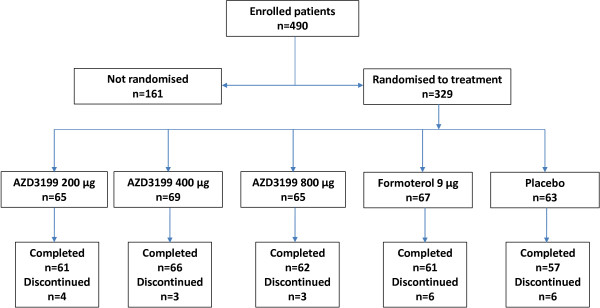
Patient flow.

At the end of the 2-week run-in period, patients were randomized to receive AZD3199 (200 μg, 400 μg or 800 μg once-daily), formoterol (9 μg twice-daily) or placebo, administered via Turbuhaler® (Figure 1). If required, a short-acting β_2_-agonist (salbutamol) could be used as reliever medication.

### Objectives, assessments and outcome measures

The primary objective of the study was to assess the efficacy of AZD3199 in patients with COPD. The primary variables were mean peak (0–4 hours post-drug administration, E_0-4_) and mean trough (24–26 hours post-drug administration, E_24-26_) forced expiratory volume in one second (FEV_1_) following morning administration after 4 weeks of treatment. Spirometry measurements were made pre-dose and then post-dose at 5 and 15 minutes and then at 1, 2, 4, 24 and 26 hours.

Forced vital capacity (FVC) (E_0–4_ and E_24–26_), patient-reported outcomes (AstraZeneca COPD symptom scores, Clinical COPD Questionnaire [CCQ], St George’s Respiratory Questionnaire for COPD [SGRQ-C]) and morning and evening use of reliever medication were assessed as secondary outcomes of the primary objective. AstraZeneca COPD symptom scores were assessed on a 5-point Likert-type scale and graded in severity from 0–4 [9]. The CCQ includes 10 questions summarized as overall mean and in three domains; Symptoms (four questions), Functional State (four questions) and Mental State (two questions). Responses were assessed on a 7-point scale from 0–6. A decrease in score of 0.4 units is regarded as the Minimal Important Difference (MID). In the SGRQ-C, each domain (symptoms, activity and impact) is scored individually and the domains are calculated by weighing the questions. Scores range from 0–100, with a change in 4 percentage units or more regarded as the MID.

The secondary objectives of the study were to investigate the safety of AZD3199 and to assess drug exposure of AZD3199. Safety variables included an examination of the nature, incidence and severity of adverse events, vital signs, ECG and laboratory assessments (haematology, clinical chemistry and urinalysis). FEV_1_ reversibility was also examined at randomization and at 26 hours after the last morning dose of AZD3199 treatment; FEV_1_ was assessed before and 15–30 minutes after inhalation of salbutamol 400 μg. After 4 weeks of treatment, blood samples were taken for analysis of AZD3199 pharmacokinetics at steady state. The maximum plasma concentration of AZD3199 (C_max_), time to C_max_ (t_max_) and the area under the plasma concentration-time curve from 0–24 hours (AUC_0–24_) were examined.

### Patients

To be eligible for the study, patients were required to be aged 40 years or above, to be symptomatic, to have a reproducible pre-bronchodilator FEV_1_ and with a diagnosis of moderate-to-severe COPD for a period of more than one year. Patients were required to have a post-bronchodilator FEV_1_ value between 40 and 80% of the predicted normal, and a post-bronchodilator FEV_1_/ FVC ratio of less than 70%. Patients were to be current or ex-smokers with a smoking history of at least 10 pack-years.

Exclusion criteria for the study included current asthma, a history of asthma or atopy (such as allergic rhinitis) before 40 years of age. Patients with any current respiratory tract disorder other than COPD, including respiratory diseases described in GOLD 2008 or JRS Guidelines 2004 as needing to be differentiated from COPD, which was considered by the investigator to be clinically significant were not included in the study. Patients with a body mass index of less than 18 kg/m^2^ or a body weight of less than 30 kg were excluded from the study, with pregnancy, lactation and past or present alcohol or drug abuse as additional criteria for exclusion. Patients with COPD exacerbations (defined as use of systemic antibiotics and/or systemic glucocorticosteroids and/or hospitalization related to COPD) in the previous 30 days, patients requiring regular oxygen use and patients with a heart condition affecting the QT/QT_c_ interval were also excluded from study participation.

### Statistical methods

The sample size calculation was based on repeated FEV_1_ assessments after 4 weeks of treatment, with peak and trough FEV_1_ as the primary endpoints. The pre-bronchodilator FEV_1_ value at the start of the treatment period constituted the baseline measurement. With 60 patients per group and a two-sided test at a 5% significance level, there was an 80% chance to detect a true difference of 5% between any two treatments. The statistical analyses were performed using SAS version 9.1. Efficacy variables were compared between treatments using analysis of variance models with treatment and country as fixed factors and baseline as a covariate. All analysis models were additive. Pairwise treatment contrasts were calculated including estimated mean differences with 95% confidence intervals and p-values. Pharmacokinetic parameters were summarized using descriptive statistics for each dose level. A closed test procedure starting with the highest dose of AZD3199 was used when determining the efficacy of AZD3199 relative to placebo.

### Ethics

The clinical study protocol was approved in writing by the independent ethics committee in each participating country. The study was performed in accordance with the ethical principles that have their origin in the Declaration of Helsinki and that are consistent with International Conference on Harmonisation (ICH)/Good Clinical Practice (GCP) and applicable regulatory requirements and the AstraZeneca policy on Bioethics. Informed consent was obtained from all patients prior to initiation of any study specific procedures, with information about the study provided to all patients at visit 1.

## Results

### Baseline characteristics

Of the 490 patients enrolled in the study, 329 were randomized to treatment. Incorrect enrolment (patients not fulfilling inclusion/exclusion criteria) and voluntary discontinuation were the primary reasons for withdrawal before randomization. The baseline characteristics of patients in each treatment arm were well-balanced and are outlined in Table 1. The average patient age was 64.1 years (range: 40–92); 75% of subjects were male; 76% were white and 24% were Asian. Participants were former (47%) or current (53%) smokers, with a median time since COPD diagnosis of 4 years (range: 0–29). The average post-bronchodilator FEV_1_ was 61% (range: 40–80) of predicted normal. Prior to enrolment inhaled corticosteroids were used by 59% of patients, with 44% of patients using LABAs, 20% using LAMAs, 63% using SABAs, 19% using SAMAs and 18% using other COPD medications, such as tolubuterol, xanthines and mucolytics. Patients were withdrawn from LABA and LAMA treatments following study enrolment. Inhaled salbutamol was permitted during the study period as reliever medication and patients who had been treated with inhaled corticosteroids (ICS) for > 30 days prior to enrolment continued to take them at a constant dose throughout the study.

**Table 1:** Patient characteristics

	**AZD3199**	**AZD3199**	**AZD3199**	**Formoterol**	**Placebo**
	**200 μg od (n = 65)**	**400 μg od (n = 69)**	**800 μg od (n = 65)**	**9 μg bid (n = 67)**	**(n = 63)**
**Gender (%)**					
Male	47 (72.3)	53 (76.8)	45 (69.2)	51 (76.1)	51 (81.0)
Female	18 (27.7)	16 (23.2)	20 (30.8)	16 (23.9)	12 (19.0)
**Age (years)**	62.4 (45–81)	63.8 (40–82)	65.3 (41–92)	64.1 (47–77)	64.8 (46–84)
**Race (%)**					
White	49 (75.4)	53 (76.8)	48 (73.8)	51 (76.1)	48 (76.2)
Asian	16 (24.6)	16 (23.2)	17 (26.2)	16 (23.9)	15 (23.8)
**BMI (kg/m**^**2**^**)**	26.6 (18–41)	25.3 (18–35)	25.3 (18–47)	25.4 (18–36)	25.7 (19–38)
**Time since COPD diagnosis (years)**^a^	4.1 (0–19)	3.1 (0–29)	3 (0–18)	4.1 (0–24)	3.5 (0–17)
**Smoking status (%)**					
Ex-smoker	34 (52.3)	32 (46.4)	27 (41.5)	36 (53.7)	27 (42.9)
Current	31 (47.7)	37 (53.6)	38 (58.5)	31 (46.3)	36 (57.1)
**Pack-years (years)**	36 (10–145)	41 (10–156)	46 (10–126)	39 (10–123)	40 (10–140)
**Total COPD symptom score**	6.0 (2–12)	5.8 (2–12)	6.0 (1–12)	5.8 (1–14)	5.7 (0–11)
**FEV**_**1 **_**(% predicted normal)**^b^	60.1 (41.4–79.8)	63.1 (39.7–79.6)	60.6 (40.4–79.9)	60.9 (40.4–79.9)	61.3 (41.7–78.5)
**Reversibility (%)**	7.4 (−11.0–27.9)	11.9 (−12.4–74.4)	10.1 (−14.1–29.6)	9.4 (−17.1–44.8)	6.8 (−7.7–51.0)
**FEV**_**1**_**/FVC (%) **^**b**^	55.2 (30.9–69.9)	56.1 (28.5–70.0)	52.4 (28.6–69.9)	54.6 (31.9–72.3)	56.1 (33.1–69.0)
**ICS use (%)**^c^	33 (50.8)	42 (60.9)	37 (56.9)	44 (65.7)	37 (58.7)
**ICS dose**^c^**(μg)**	737.6 (320–1000)	638.1 (200–1000)	620.5 (320–2000)	649.3 (200–1500)	671.1 (250–1000)
**LAMA use (%)**^d^	10 (15.4)	11 (15.9)	19 (29.2)	11 (16.4)	16 (25.4)
**LABA use (%)**^e^	29 (44.6)	30 (43.5)	29 (44.6)	28 (41.8)	28 (44.4)
**SAMA use (%)**^**f**^	14 (21.5)	16 (23.2)	9 (13.8)	9 (13.4)	14 (22.2)
**SABA use (%)**^**g**^	41 (63.1)	44 (63.8)	40 (61.5)	41 (61.2)	41 (65.1)
**Other COPD medication**^**h**^	13 (20.0)	10 (14.5)	10 (15.4)	15 (22.4)	10 (15.9)

^a^ Median; ^b^ Post-bronchodilator; ^c^ Inhaled corticosteroids; ^d^ Long-acting muscarinic antagonist; ^e^ Long acting β_2_-agonist; ^f^ Short-acting muscarinic antagonist; ^g^ Short-acting β_2_-agonist; ^h^ Includes tolubuterol, xanthines and mucolytics.

Following randomization, 22 patients discontinued the study, primarily due to exacerbations of COPD, which fulfilled protocol-specific withdrawal criteria (n = 7). Additional reasons for patient withdrawal included incorrect enrolment (n = 5), voluntary discontinuation (n = 5), the development of an adverse event (n = 3), non-compliance to protocol (n = 1), with one patient lost to follow-up.

### Objectives, assessments and outcome measures

#### Peak and trough FEV_1_

**Figure 2:**
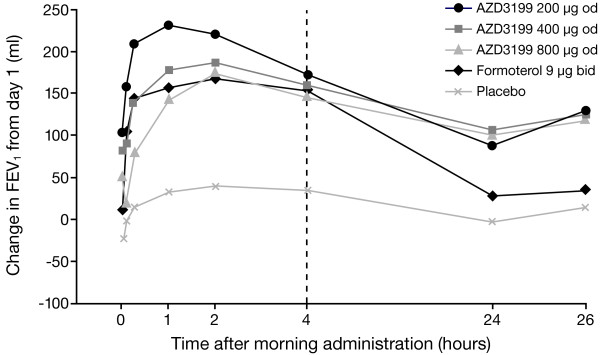
**Adjusted mean changes in FEV**_**1 **_**after 4 weeks of treatment.** Values shown are FEV_1_ changes (from baseline [day 1] to end of week 4) at each post-dose time-point. od = once daily; bid = twice daily. The formoterol study group received additional study medication at 12 hours.

After 4 weeks of treatment, all doses of AZD3199 had increased mean peak and trough FEV_1_ values compared with placebo, but no clear dose–response was observed (Figure 2); peak FEV_1_ increased by 106–171 ml with AZD3199 treatment compared with placebo, while trough FEV_1_ increased by 97–110 ml (statistically significant at all dose levels).

Formoterol 9 μg twice-daily treatment statistically significantly increased peak FEV_1_ (114 ml increase) but not trough FEV_1_ (18 ml increase).

All doses of once-daily AZD3199 had a comparable effect to formoterol 9 μg twice-daily with regards to peak FEV_1_. On trough FEV_1_ there was a tendency towards increased bronchodilation for all doses of AZD3199 versus formoterol 9 μg twice-daily (79–93 ml increase), but no statistically significant differences were observed (p > 0.05) (Table 2).

**Table 2:** **Treatment estimates and pairwise contrasts for FEV**_**1 **_**and FVC E**_**0-4 **_**and E**_**24-26**_**, additive model (ml)**

	**Treatment**	**Mean change**	**Treatment contrasts**			
				**Difference (ml)**	**95% CI**	P-value*
**FEV**_**1**_**, E**_**0–4**_	AZD3199 200 μg od	199.7	AZD3199 200 vs Placebo	171	69.2, 272.9	0.001
	AZD3199 400 μg od	163.2	AZD3199 400 vs Placebo	135	34.7, 234.3	0.008
	AZD3199 800 μg od	134.3	AZD3199 800 vs Placebo	106	4.6, 206.8	0.041
	Formoterol 9 μg bid	142.8	Formoterol 9 vs Placebo	114	12.8, 215.6	0.027
	Placebo	28.6	AZD3199 200 vs Formoterol 9	57	−42.7, 156.4	0.262
			AZD3199 400 vs Formoterol 9	20	−77.4, 118.2	0.682
			AZD3199 800 vs Formoterol 9	−8	−107.2, 90.3	0.866
**FEV**_**1**_**, E**_**24–26**_	AZD3199 200 μg od	104.1	AZD3199 200 vs Placebo	99	2.4, 194.9	0.045
	AZD3199 400 μg od	115.9	AZD3199 400 vs Placebo	110	16.0, 204.7	0.022
	AZD3199 800 μg od	102.1	AZD3199 800 vs Placebo	97	0.5, 192.5	0.049
	Formoterol 9 μg bid	23.4	Formoterol 9 vs Placebo	18	−78.0, 113.7	0.714
	Placebo	5.5	AZD3199 200 vs Formoterol 9	81	−13.4, 174.9	0.092
			AZD3199 400 vs Formoterol 9	93	0.1, 185.0	0.050
			AZD3199 800 vs Formoterol 9	79	−15.0, 172.4	0.100
**FVC, E**_**0–4**_	AZD3199 200 μg od	186.3	AZD3199 200 vs Placebo	192	50.2, 334.3	0.008
	AZD3199 400 μg od	198.2	AZD3199 400 vs Placebo	204	64.9, 343.4	0.004
	AZD3199 800 μg od	146.7	AZD3199 800 vs Placebo	153	11.7, 293.5	0.034
	Formoterol 9 μg bid	162.1	Formoterol 9 vs Placebo	168	26.5, 309.5	0.020
	Placebo	−5.9	AZD3199 200 vs Formoterol 9	24	−114.7, 163.2	0.731
			AZD3199 400 vs Formoterol 9	36	−100.0, 172.3	0.602
			AZD3199 800 vs Formoterol 9	−15	−153.1, 122.3	0.826
**FVC, E**_**24–26**_	AZD3199 200 μg od	69.8	AZD3199 200 vs Placebo	86	−43.3, 214.9	0.192
	AZD3199 400 μg od	167.9	AZD3199 400 vs Placebo	184	57.4, 310.5	0.005
	AZD3199 800 μg od	97.9	AZD3199 800 vs Placebo	114	−14.7, 242.5	0.082
	Formoterol 9 μg bid	13.5	Formoterol 9 vs Placebo	30	−99.1, 158.1	0.652
	Placebo	−16.0	AZD3199 200 vs Formoterol 9	56	−70.0, 182.6	0.381
			AZD3199 400 vs Formoterol 9	155	30.7, 278.2	0.015
			AZD3199 800 vs Formoterol 9	84	−41.2, 210.1	0.187

*Not adjusted for multiple comparisons.

#### FVC

In a similar manner to FEV_1_, peak FVC was statistically significantly increased with all doses of AZD3199 compared with placebo, but no clear dose–response was observed (153–204 ml increase). Trough FVC was increased by 86–184 ml with AZD3199 compared with placebo, but the difference was not statistically significant for AZD3199 200 μg or 800 μg.

Both peak and trough FVC were increased following treatment with formoterol 9 μg twice-daily (168 ml and 30 ml respectively), but this only reached statistical significance for peak FVC (p = 0.02).

There was no statistically significant difference in peak FVC between AZD3199 once-daily and formoterol 9 μg twice-daily (Table 2).

#### Patient-reported outcomes

Overall mean CCQ scores (Table 3) were statistically significantly reduced in the AZD3199 800 μg and formoterol 9 μg twice-daily treatment groups, in comparison with placebo (p = 0.016 and 0.018 respectively). When assessed by sub-domain, AZD3199 800 μg statistically significantly reduced scores for mental state (p = 0.016).

**Table 3:** Treatment comparisons for changes from run-in to treatment

	**Treatment**	**Mean change**	**Treatment contrasts**			
				**Difference**	**95% CI**	P-value*
**CCQ variable**						
Overall mean	AZD3199 200 μg od	−0.38	AZD3199 200 vs Placebo	−0.262	−0.457, -0.067	0.009
	AZD3199 400 μg od	−0.30	AZD3199 400 vs Placebo	−0.178	−0.369, 0.014	0.068
	AZD3199 800 μg od	−0.36	AZD3199 800 vs Placebo	−0.240	−0.435, -0.045	0.016
	Formoterol 9 μg bid	−0.36	Formoterol 9 vs Placebo	−0.234	−0.427, -0.040	0.018
	Placebo	−0.12	AZD3199 200 vs Formoterol 9	−0.028	−0.219, 0.162	0.770
			AZD3199 400 vs Formoterol 9	0.056	−0.132, 0.243	0.559
			AZD3199 800 vs Formoterol 9	−0.007	−0.198, 0.184	0.945
**AZ Diary card COPD Symptoms**						
Total Score (0–16)	AZD3199 200 μg od	−0.94	AZD3199 200 vs Placebo	−0.480	−1.011, 0.051	0.076
	AZD3199 400 μg od	−0.94	AZD3199 400 vs Placebo	−0.473	−1.000, 0.054	0.078
	AZD3199 800 μg od	−1.19	AZD3199 800 vs Placebo	−0.723	−1.254, -0.192	0.008
	Formoterol 9 μg bid	−0.62	Formoterol 9 vs Placebo	−0.152	−0.685, 0.380	0.574
	Placebo	−0.46	AZD3199 200 vs Formoterol 9	−0.327	−0.852, 0.197	0.220
			AZD3199 400 vs Formoterol 9	−0.321	−0.841, 0.200	0.226
			AZD3199 800 vs Formoterol 9	−0.570	−1.095, -0.046	0.033
**SGRQ-C Scores**						
Total Score	AZD3199 200 μg od	−5.61	AZD3199 200 vs Placebo	−3.01	−7.31, 1.28	0.169
	AZD3199 400 μg od	−8.18	AZD3199 400 vs Placebo	−5.59	−9.78, -1.40	0.009
	AZD3199 800 μg od	−4.63	AZD3199 800 vs Placebo	−2.03	−6.29, 2.23	0.350
	Formoterol 9 μg bid	−4.93	Formoterol 9 vs Placebo	−2.33	−6.65, 1.99	0.289
	Placebo	−2.60	AZD3199 200 vs Formoterol 9	−0.68	−4.89, 3.53	0.751
			AZD3199 400 vs Formoterol 9	−3.25	−7.34, 0.83	0.118
			AZD3199 800 vs Formoterol 9	0.30	−3.86, 4.47	0.886

*Not adjusted for multiple comparisons.

AZ, AstraZeneca; CCQ, Clinical COPD Questionnaire; SGRQ-C, St George’s Respiratory Questionnaire for COPD.

All doses of AZD3199 reduced COPD symptom scores, and this reached statistical significance for AZD3199 800 μg in reducing total symptom score (p = 0.008) (Table 3) and the individual symptom scores for breathlessness (p = 0.016), chest tightness (p = 0.05) and night-time awakenings (p = 0.003), in comparison with placebo. Total symptom score and individual symptom score were not significantly altered in the formoterol 9 μg twice-daily treatment group versus placebo. In comparison with formoterol 9 μg twice-daily, AZD3199 800 μg also statistically significantly reduced total symptoms (p = 0.033) and breathlessness (p = 0.007).

All active treatments reduced total SGRQ-C scores versus placebo, with scores lowered by 2.03–5.59 units with AZD3199 treatment (not statistically significant for AZD3199 800 μg) and 2.33 units for formoterol 9 μg twice-daily (Table 3). A decrease of 4 units is regarded as the clinically relevant MID. Reductions in the sub-domain scores for symptoms, activity and impact were also observed. However, reductions in total and SGRQ sub-domain scores did not reach statistical significance for AZD3199 800 μg and formoterol 9 μg, in comparison with placebo.

All doses of AZD3199 statistically significantly reduced the daily use of rescue medication in comparison with the placebo group, with the greatest reduction in rescue medication use after AZD3199 400 μg and 800 μg. In contrast, formoterol 9 μg twice-daily did not statistically significantly influence the daily use of rescue medication when compared with placebo.

#### FEV_1_ reversibility

In all AZD3199 treatment groups, FEV_1_ measurements post-salbutamol inhalation increased by approximately 50 ml over the course of the study. Numerically, but not statistically, larger increases in post-salbutamol FEV_1_ values were observed for the AZD3199 treatment arms than for the placebo and formoterol 9 μg treatment arms. The increase in pre-salbutamol FEV_1_ was approximately 100 ml for all AZD3199 treatment groups, resulting in a numerically smaller (5%) reversibility compared with the groups receiving formoterol 9 μg twice-daily or placebo. Thus, there were no adverse effects of AZD3199 on the acute bronchodilatory action of salbutamol.

### Safety

In the placebo group, one death was reported but this was judged as not causally related to the study drug, and was attributed to circulatory collapse. Serious adverse events were evenly distributed between the treatment groups. Overall, 8 patients experienced 10 serious adverse events (AZD3199 200 μg, n = 1; AZD3199 400 μg, n = 4; AZD3199 800 μg, n = 0; formoterol 9 μg, n = 3; placebo, n = 2), of which 2 (both in the formoterol 9 μg treatment group) were considered to be causally related to the study medication. Discontinuation of treatment due to adverse events (DAEs) was reported for 11 patients, primarily due to exacerbation of COPD. DAEs were evenly distributed across the treatment groups (AZD3199 200 μg, n = 0; AZD3199 400 μg, n = 2; AZD3199 800 μg, n = 2; formoterol 9 μg, n = 3; placebo, n = 4).

Altogether, 128 adverse events were experienced by 84 patients (26%). The majority of adverse events (95.3%) were considered to be of mild or moderate intensity, with nasopharyngitis, cough, COPD exacerbation and throat irritation reported most frequently (Table 4). The number of patients experiencing an adverse event was highest in the group receiving AZD3199 400 μg and lowest in the group receiving AZD3199 200 μg. The higher incidence of adverse events in the AZD3199 400 μg treatment group was attributable to several patients experiencing multiple events. Less than one-third (31.3%) of the total adverse events were considered to be causally related to the study medication.

**Table 4:** Adverse events

	**Number (%) of patients**					
**Preferred term**	**AZD3199**	**AZD3199**	**AZD3199**	**Formoterol**		**Total (n = 329)**
	**200 μg od (n = 65)**	**400 μg od (n = 69)**	**800 μg od (n = 65)**	**9 μg bid (n = 67)**	**Placebo (n = 63)**	
Patients with any adverse event	8 (12.3)	21 (30.4)	19 (29.2)	18 (26.9)	18 (28.6)	84 (25.5)
Nasopharyngitis	1 (1.5)	4 (5.8)	3 (4.6)	2 (3.0)	3 (4.8)	13 (4.0)
Cough	0 (0.0)	4 (5.8)	4 (6.2)	2 (3.0)	0 (0.0)	10 (3.0)
Chronic obstructive pulmonary disease	1 (1.5)	2 (2.9)	2 (3.1)	2 (3.0)	2 (3.2)	9 (2.7)
Throat irritation	1 (1.5)	0 (0.0)	1 (1.5)	2 (3.0)	1 (1.6)	5 (1.5)
Bronchitis	0 (0.0)	0 (0.0)	2 (3.1)	0 (0.0)	2 (3.2)	4 (1.2)
Headache	0 (0.0)	2 (2.9)	1 (1.5)	1 (1.5)	0 (0.0)	4 (1.2)
Product taste abnormal	1 (1.5)	0 (0.0)	3 (4.6)	0 (0.0)	0 (0.0)	4 (1.2)

There were no consistent clinically significant changes across treatment groups in blood pressure, pulse, ECG or clinical laboratory parameters. As such, no safety concerns were raised for AZD3199.

### Pharmacokinetics

Analysis of C_max_ and AUC_0–24_ for AZD3199 indicated steady-state exposure to be dose-proportional (Table 5). The exposure of AZD3199 was approximately half of that previously observed in healthy volunteers and patients with mild-to-moderate asthma. Absorption of AZD3199 was rapid, with a median t_max_ of 15 minutes for all doses of AZD3199.

**Table 5:** Pharmacokinetics of AZD3199

**Variable**	**Dose group**	**n**	**G mean**^**a**^	**CV**^**b**^
C_max_ (nmol/L)	200 μg	53	1.136	118.5
	400 μg	64	1.882	190.1
	800 μg	59	4.011	107.0
AUC_0-24_ (nmol*h/L)	200 μg	53	4.91	99.5
	400 μg	64	8.75	138.7
	800 μg	59	14.85	107.1

^a^Geometric mean; ^b^Coefficient of variation.

## Discussion

This is the first study to evaluate the efficacy and safety of AZD3199 in patients with COPD. Treatment doses were selected based on the results of a Phase IIa study in patients with asthma (NCT00736489) and a multiple ascending dose (MAD) study in healthy volunteers (NCT00713271), where AZD3199 was administered via Turbuhaler®. The low dose of 200 μg was selected based on the Phase IIa study, as the lowest dose with a potential for 24-hour duration of action. To provide a 2-fold safety margin to the maximum dose (1680 μg) given to healthy volunteers in the MAD study, the highest dose in this study was chosen as 800 μg.

All doses of AZD3199 produced effective bronchodilation with 24-hour duration of action that was comparable to, or greater than, that achieved with formoterol twice-daily. However, no clear dose–response was observed for the bronchodilatory effects of AZD3199 at either peak or trough effects. Due to the lack of a clear dose–response, the present study was unable to identify a definitive dose of AZD3199 to carry forward to Phase III investigation. The current data suggest that in COPD patients a plateau of the dose–response curve may have been reached at or below a once-daily dose of AZD3199 200 μg. The effects of AZD3199 at steady state may be enhanced in patients with COPD relative to single doses, resulting in lower doses being sufficient to achieve a maximal bronchodilator response. Doses of AZD3199 at less than 200 μg may therefore be sufficient to maintain bronchodilation over 24 hours in patients with COPD.

A reduction in COPD symptom scores was seen after AZD3199 800 μg and a reduction in the daily use of reliever medication was seen after all doses of AZD3199, in comparison with placebo. Although the present study was not powered to look at improvements in patient-reported outcomes, some evidence of positive effects on these variables was observed. CCQ scores were reduced with AZD3199 200 μg, providing support for the use of this dose in Phase III investigation. SGRQ-C scores were also improved, complementing the bronchodilating effect achieved. It has been suggested that regular administration of LABAs can lead to the development of tolerance to the effects of bronchodilation, potentially reducing the response to reliever medications such as salbutamol. Tolerance did not appear to develop to AZD3199 or formoterol in this 4-week study, as there was no adverse effect on the post-salbutamol FEV_1_ levels.

The incidence of adverse effects was generally low and similar between study groups, and most adverse effects were of mild intensity. There were few cases of post-inhalation cough in the present study, but somewhat more cases with AZD3199 400 μg and 800 μg. There were no clinically significant adverse findings for vital signs, laboratory parameters and ECGs; AZD3199 was therefore considered to be well-tolerated and no safety concerns were raised. A limitation of this study was that the relatively short 4-week duration of the study precluded examination of longer term safety and tolerability parameters, but these can be assessed in future studies. Four weeks is generally considered adequate for assessments of efficacy and dose response.

Analysis of plasma AZD3199 revealed dose-proportional pharmacokinetics. However, systemic exposure to AZD3199 in patients with COPD was approximately half of that previously reported in healthy volunteers and patients with mild-to-moderate asthma, which may be related to reduced absorption or increased drug metabolism. This effect is in line with previous studies demonstrating reduced systemic exposure to formoterol, budesonide and fluticasone in patients with COPD, compared with healthy volunteers [10].

## Conclusions

All of the AZD3199 doses produced 24-hour bronchodilation, but with no clear dose–response, suggesting that doses of 200 μg or less may be sufficient to maintain bronchodilation over 24 hours in patients with COPD. No safety concerns were identified. Further studies are required to determine the appropriate once-daily AZD3199 dose for COPD.

## Abbreviations

COPD: Chronic obstructive pulmonary disease.

## Competing interests

FM has received research grants for participating in multicenter trials sponsored by AstraZeneca and has also received fees for speaking at conferences sponsored by Boehringer Ingelheim, Pfizer and GlaxoSmithKline. He has served on an advisory board for GlaxoSmithKline and Boehringer Ingelheim and received research grants for participating in multicenter trials sponsored by GlaxoSmithKline, Boehringer Ingelheim, Altana Pharma, Merck, Nycomed and Novartis. He has received unrestricted research grants from Boehringer Ingelheim and GlaxoSmithKline. He holds a CIHR/GSK research chair on COPD.

OB, CJ and TB were employees of AstraZeneca at the time this study was conducted and during the development of this manuscript and all have shares in AstraZeneca.

PK has received research grants for participating in multicenter trials sponsored by AstraZeneca and has received reimbursement for attending a symposium, fees for speaking and consultation fees from Adamed, Allergopharma, AstraZeneca, Boehringer Ingelheim, GSK, Krka, MSD, Novartis, Nycomed, Pfizer, Sandoz, Sanofi Aventis, Stallergen, Teva, UCB.

TS has received research grants for participating in multicenter trials sponsored by AstraZeneca and has received lecture fees from Novartis Pharma KK, Astellas Pharma Inc., MSD KK, Shionogi & Co. Ltd and Dainippon Sumitomo Pharma Co. Ltd.

VT has received research grants for participating in multicenter trials sponsored by AstraZeneca.

YI has has received research grants for participating in multicenter trials sponsored by AstraZeneca.

This study was supported by AstraZeneca.

## Authors’ contributions

PK, YI, VIT, TS, OB and FM participated in the design, recruitment and/or conduct of the study and were involved at all stages in the development of the manuscript. TB participated in the design of the study and performed the statistical analysis. CJ is the AstraZeneca physician responsible for AZD3199. All authors read and approved the final manuscript.
